# Aggressive giant prolactinoma: a case report

**DOI:** 10.1186/s13256-022-03390-y

**Published:** 2022-04-30

**Authors:** Marisa Khatijah Borhan, Florence Hui Sieng Tan

**Affiliations:** Endocrinology Unit, Medical Department, Sarawak General Hospital, Kuching Sarawak, Malaysia

**Keywords:** Giant prolactinoma, Aggressive prolactinoma, Temozolomide, Case report

## Abstract

**Background:**

Managing treatment-resistant aggressive giant prolactinoma can be challenging, as the diagnosis is often complex, and treatment beyond dopamine agonists, surgery, and radiotherapy is limited.

**Case presentation:**

A 21-year-old Malay woman first presented to our hospital at the age of 16 years with 1-year history of reduced vision and 2 years of amenorrhea. Her baseline prolactin level was 255,894 µIU/mL with secondary hypogonadism, and pituitary magnetic resonance imaging revealed a giant prolactinoma (2.8 × 3.2 × 4.2 cm^3^) with suprasellar extension and optic chiasmal compression. She was initially treated with cabergoline, and reductions in the prolactin level and tumor mass were achieved, leading to vision improvement and resumption of normal menstruation. However, she developed recurrent tumor growth and hyperprolactinemia, causing relapse of symptoms, and she needed surgery. Eventually, despite three tumor debulking surgeries and escalation of cabergoline doses up to 1 mg/day, her tumor progressed with aggressive characteristics. Following a multidisciplinary meeting, the patient is initiated on temozolomide therapy after considering the long-term side effects of radiotherapy in her case.

**Conclusion:**

This case highlights the importance of early identification of treatment-resistant prolactinoma and the need for a multidisciplinary approach in managing aggressive prolactinoma in young patients, particularly regarding timely implementation of temozolomide therapy.

## Background

Prolactinoma is the most common pituitary tumor, accounting for up to 40% of all functioning pituitary adenomas. Giant prolactinoma, defined as a prolactinoma larger than 4 cm with secreted prolactin levels greater than 1000 µg/L, is rare and constitutes only approximately 1–5% of prolactin-secreting tumors [1]. Due to their large size, giant prolactinomas tend to invade adjacent structures, especially the suprasellar and cavernous sinuses, causing compressive mass effects, visual disturbances, cranial nerve palsies, and hypopituitarism. Nevertheless, prolactinomas, including giant prolactinomas, are exceptional pituitary tumors that respond well to dopamine agonists (DAs). A review of 13 case series involving 97 patients with giant prolactinomas treated with DA reported normalization of prolactin levels in 60%, a significant reduction (> 30%) in tumor size in 83%, and improvements in visual symptoms in 96% of those who presented with visual field defects [2].

DA resistance has, however, been reported in approximately 10% of cabergoline-treated prolactinomas, with higher prevalence seen in males, children, adolescents, and those with larger tumors (macroprolactinoma and giant prolactinoma), invasive tumors, cystic tumors, and tumors with MEN1 and AIP mutations [3]. Aggressive prolactinomas are even rarer and exhibit aggressive features characterized by clinically relevant tumor growth or multiple recurrences despite optimal standard therapies combining medical, surgical, and radiotherapy treatment approaches [4]. We report a rare case of giant prolactinoma that showed secondary DA resistance and aggressive behaviors. This case report highlights the challenges in the diagnosis of aggressive prolactinoma and management of DA-resistant aggressive giant prolactinoma, especially in the setting of limited treatment modalities.

## Case presentation

A 21-year-old Malay female patient first presented at the age of 16 years with 1-year history of blurred vision and 2 years of amenorrhea. Clinically, she had bitemporal hemianopia and visual acuity limited to finger counting. She had no cushingoid or acromegaly features. Her baseline prolactin level was 255,894 µIU/mL with low estradiol (11.62 pg/mL) and normal FSH (6.55 IU/L). Her free T4 (18.44 pmol/L) and morning cortisol levels (11.52 µg/dL) were within normal limits. Pituitary MRI revealed a giant prolactinoma (2.8 × 3.2 × 4.2 cm^3^) with suprasellar extension and optic chiasmal compression. Cabergoline was initiated at 0.5 mg thrice weekly. While on cabergoline, her prolactin level dropped to 9420 µIU/mL (96% reduction) and her vision improved (visual acuity; VA: right eye 6/24, left eye 6/18), with resolved bitemporal hemianopia. She also reported resumption of normal menstruation. Repeated pituitary MRI showed a significant reduction in tumor size (2.1 × 1.7 × 2.9 cm^3^).

**Fig. 1:**
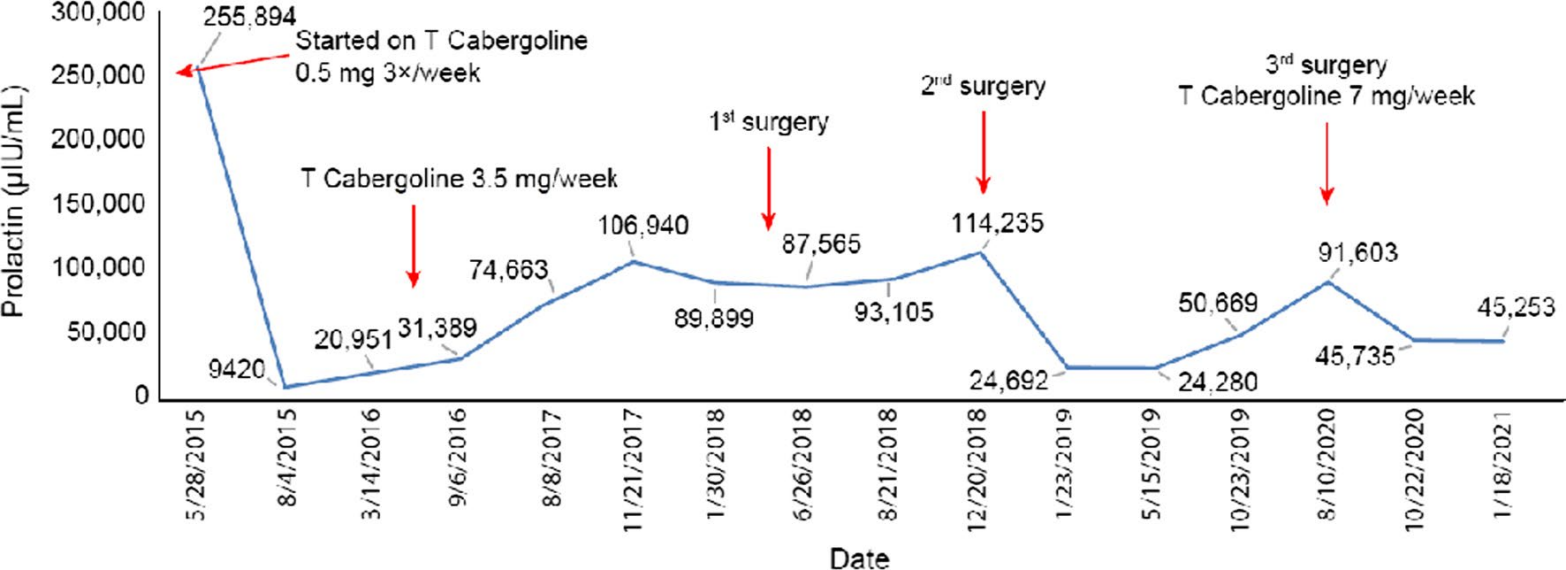
Trend of prolactin level

However, despite adhering to the treatment, she never achieved normoprolactinemia. PEG precipitation for macroprolactin ruled out macroprolactinemia with 99% recovery. After 11 months of treatment, her prolactin continued to rise despite uptitration of her cabergoline to 3.5 mg weekly (Fig. 1). A repeat pituitary MRI showed an increased tumor size (2.5 × 2.5 × 3.4 cm^3^), with the tumor compressing the hypothalamus, while the optic chiasm and pituitary stalk were not visualized. She was otherwise asymptomatic. Failing medical therapy, she underwent transsphenoidal surgery (TSS), whereby intraoperatively, 30% of the tumor at the suprasellar anterior part was unresectable, as the tumor was tough and adherent to the underlying structure. Histopathology examination (HPE) revealed an atypical pituitary adenoma with staining showing extensive perivascular and interstitial fibrosis, mitotic activity of 8/10 HPF, and Ki-67 proliferative index of 4–5%.

**Fig. 2:**
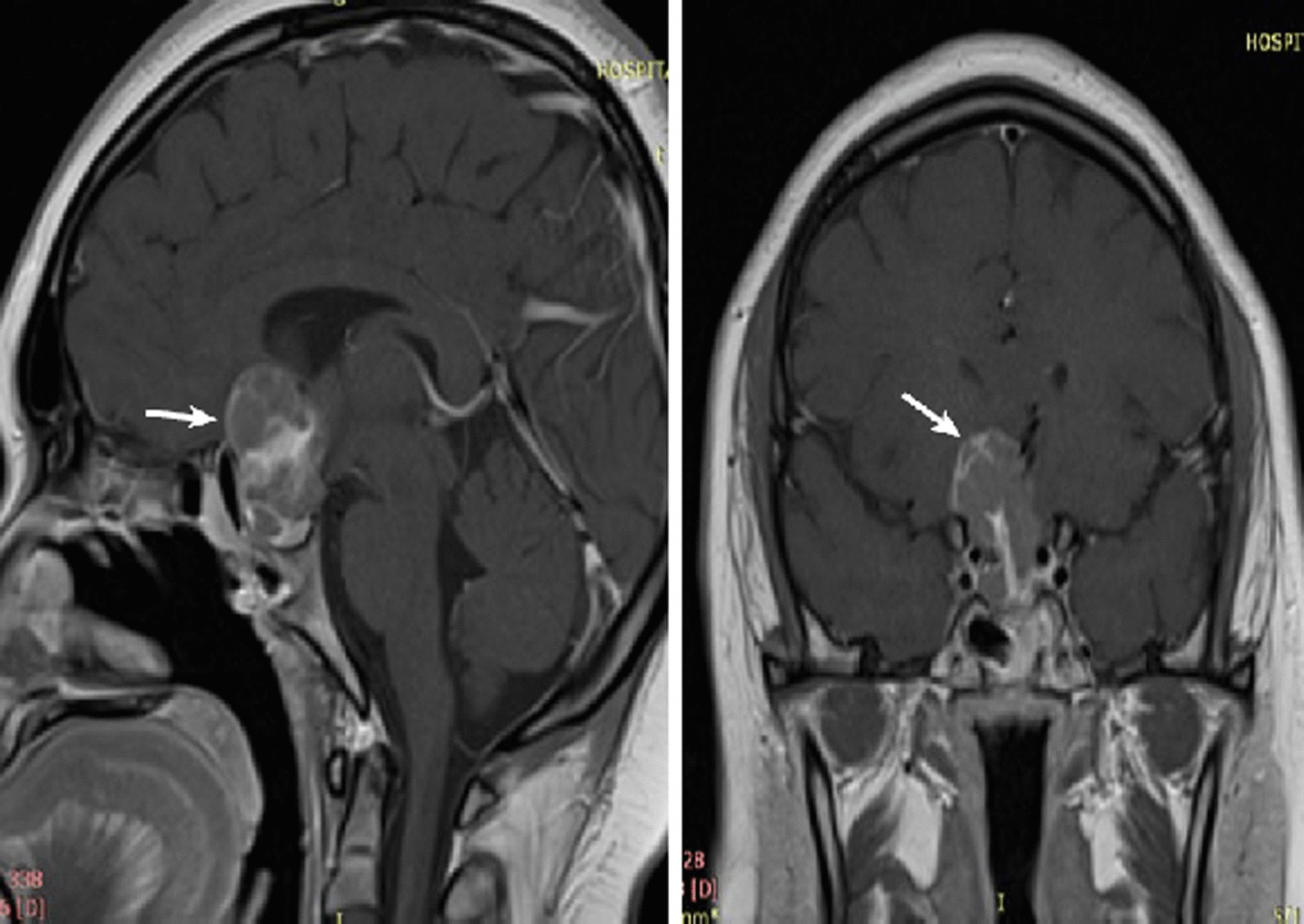
Postoperative sagittal and coronal T1-weighted magnetic resonance imaging pituitary performed after the first transsphenoidal surgery. The magnetic resonance imaging revealed a pituitary lesion with residual mass (size 3.5 × 3.0 × 2.7 cm^3^) seen mainly in the right suprasellar region (white arrow)

Under close monitoring, her prolactin remained elevated despite cabergoline dosage of 3.5 mg/week. Her menstruation ceased, and she developed recurrent right temporal hemianopia and worsening VA. The pituitary MRI (Fig. 2) showed an increased tumor size (3.5 × 3.0 × 2.7 cm^3^) with a prolactin level of 114,235 µIU/mL. She underwent a second TSS procedure with postoperative HPE staining that was positive for synaptophysin and negative for GFAP, EMA, and NB84. The mitotic activity was 2–3/10 HPF, and the Ki-67 proliferative index was > 3–4%. Postoperatively, her prolactin dropped to 24,692 µIU/mL. However, she remained amenorrheic and was started on hormone replacement therapy as well as thyroxine replacement due to the development of TSH deficiency (free T4 10.83 pmol/L).

**Fig. 3:**
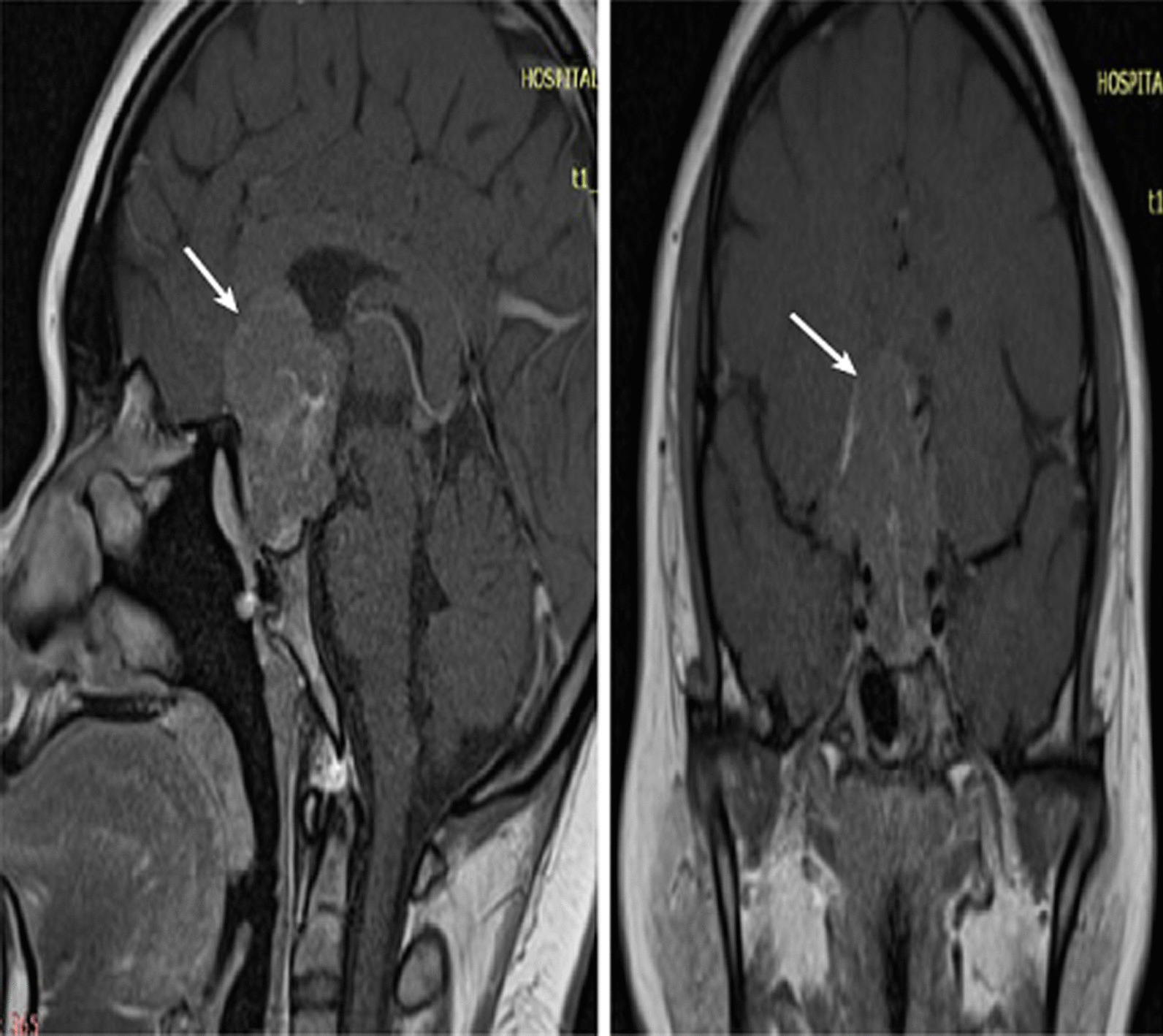
Preoperative sagittal and coronal T1-weighted magnetic resonance imaging pituitary performed prior to the third debulking surgery. MRI revealed an increased tumor size (2.6 × 3.0 × 3.7 cm^3^) with a larger suprasellar component (white arrow) compressing towards the corpus callosum, lateral ventricle, and third ventricle

Unfortunately, her disease continued to progress, showing an increased tumor size (2.6 × 3 × 3.7 cm^3^) with larger sellar and suprasellar components and mass effects (Fig. 3). Her prolactin level remained high (91,603 µIU/mL) despite being on cabergoline 0.75 mg daily, which was further titrated up to 1 mg daily. Her visual impairment worsened with bitemporal hemianopia and right RAPD. A decision was made to perform a third surgery via a transcranial approach to gain better access to the suprasellar portion of the tumor. Intraoperatively, there was remnant of the tumor in the right superior region extended towards the hypothalamus and the third ventricular floor, which was unresectable due to firm adherence to the surrounding brain.

Postoperative pituitary MRI showed a smaller pituitary mass measuring 2.6 × 2.2 × 3.0 cm^3^, and prolactin levels were stable at 45,000 µIU/mL on cabergoline 1 mg daily. A multidisciplinary team meeting with neurosurgery, radiology, oncology, and endocrinology teams was conducted. Considering the patient’s young age, the proximity of the tumor to the left optic nerve, and the resistance of the atypical pituitary adenoma to both medical and surgical therapy, which suggested tumor aggressiveness, the patient is planned for temozolomide (TMZ) therapy with a view for radiotherapy pending assessment of the tumor’s response to TMZ. TMZ (220 mg daily for 5 days per month) was initiated in November 2021 for six cycles. Aside from mild nausea, she tolerated the TMZ therapy well. Three months post TMZ therapy, her prolactin level slightly reduced (40,912 µIU/mL), and she denied any headache or blurring of vision and has regular menstruation.

## Discussion

Aggressive prolactinomas with invasion into the surrounding and remote structures can cause challenges in treatment. Our case shows a combination of features suggestive of an aggressive clinical course, including an invasive giant prolactinoma, high Ki-67 index, rising prolactin level, and recurrent tumor growth despite high-dose DA treatment and multiple surgeries. While giant prolactinomas are almost always invasive and thus associated with a lower cure rate and a higher recurrence rate than noninvasive adenomas, most of them are still sensitive to DA treatment, which is the primary therapy even in the presence of visual or neurological symptoms [2, 5]. As seen in our patient, her prolactin level and tumor size responded well after she was started on cabergoline. Unfortunately, during the course of her disease, she developed secondary resistance to cabergoline, and her tumor continued to progress with aggressive characteristics.

It is important to note that tumor size, invasiveness, or resistance to DA alone do not equate to tumor aggressiveness. Even with a “massive” giant prolactinoma > 6 cm with extrasellar extension, a patient can achieve biochemical and clinical remission with a combination of medical therapy and surgery [5]. The 2017 WHO classification of tumors of the pituitary gland considers tumor cell lineages, proliferative potential (mitotic count and Ki-67 index), and tumor invasion when identifying aggressive tumors. Rapidly growing pituitary tumors with radiological invasion and high Ki-67 proliferation index are classified as high-risk pituitary tumors; hence, patients with a combination of these features should undergo intensive investigation and close monitoring. The 2018 European Society of Endocrinology (ESE) clinical practice guidelines for the management of aggressive pituitary tumors and carcinomas recommend that the diagnosis of an aggressive prolactinoma be considered in patients with radiologically invasive and rapidly growing tumors or clinically relevant tumor growth despite optimal standard therapies [4]. It further recommends an evaluation of the Ki-67 index and p53 immunodetection and mitotic count evaluation when the Ki-67 index is ≥ 3%. Ki-67 ≥ 3% is the most frequent positive marker in aggressive pituitary tumors [4]. In our case, HPE of the tumor repeatedly reported an atypical adenoma with elevated Ki-67 index of ≥ 3% and increased mitotic activity > 2.

Unlike DA-resistant prolactinoma, the exact incidence of aggressive prolactinoma is not known, likely due to limited case reports and a lack of universal terminology. In a multicenter study of 92 patients with cabergoline-resistant prolactinomas, only four patients were identified as having locally aggressive tumors [3]. These four patients were documented to have macroadenoma, invasion into adjacent structures (suprasellar extension, cavernous sinus invasion), and demonstrated tumor progression despite cabergoline dosages up to 8 mg/week, multiple pituitary surgeries, and radiotherapy. In a cohort study by Ceccato *et al.*, 102 out of the 582 patients studied had aggressive pituitary tumors defined based on their radiological features (tumor size, local invasion), pathological reports (MIB-1 > 3%, p53 immunoreactivity, increased mitotic activity), and response to treatment. Twenty-three out of 102 of these aggressive pituitary adenomas were prolactin-secreting tumors [6].

Management of aggressive prolactinoma requires multidisciplinary care. Debulking surgery performed by experienced neurosurgeons should be considered in patients with compressive symptoms such as acute loss of vision or intractable headache despite DAs. Surgery, however, is rarely curative in giant and invasive prolactinomas [7], since tumor invasion, especially into the cavernous sinus, is one of the contributing factors for incomplete tumor resection [8, 9]. In addition, intratumoral fibrosis resulting from long-term DA treatment can further complicate surgical resection, as occurred in our patient, where the tumor remnant was found to be fibrotic and adherent to surrounding brain tissues despite application of the transcranial approach to obtain better access to tumors that extend into the suprasellar region. While radiation therapy, either fractionated external-beam radiation therapy or stereotactic radiosurgery, can be considered for patients with clinically significant tumor growth despite standard medical therapy and surgery, its risks and long-term side effects, including optic and cranial neuropathy, secondary brain tumors such as meningioma, cerebrovascular disease, and hypopituitarism, must be considered thoroughly, especially in young patients [4].

In recent years, more data have supported the use of TMZ for aggressive pituitary tumors that fail to respond to other treatment modalities. TMZ is an oral chemotherapeutic agent that readily crosses the blood–brain barrier, and its use in aggressive prolactinoma has shown positive outcomes, including the disappearance of metastases, primary tumor reduction, and normalization of prolactin. An analysis of a case series by Halevy *et al.* showed that functioning tumors, especially prolactinomas and corticotroph tumors, responded well to TMZ compared with nonfunctioning tumors [10]. A systematic review of TMZ use in aggressive prolactinoma that is resistant to standard therapy showed that, out of 23 cases of aggressive prolactinomas treated with TMZ, reduction  of tumor size of varying extents was seen in 15 patients (79%), with 1 patient having tumor stabilization and 3 patients (15.8%) having tumor progression. The mean reduction in prolactin levels was 89%, and treatment with TMZ is generally well tolerated [11]. In 2016, a study on the outcome of 157 patients with aggressive pituitary tumors and carcinoma receiving TMZ treatment, either as monotherapy or in combination, found that, in the 116 patients with aggressive pituitary tumors, complete regression of the disease was seen in 5 patients (4%), which was never previously reported for treatments using conventional cytotoxic drugs [12]. Based on these studies which reported its efficacy with good tolerability and few side effects, the 2018 ESE guidelines recommend TMZ as first-line chemotherapy for aggressive prolactinoma and carcinoma [4].

## Conclusion

Aggressive giant prolactinoma is a challenging clinical scenario, as treatment modalities beyond DAs, surgery, and radiotherapy are limited. When treatment fails and the tumors persistently cause significant complications, TMZ is a promising treatment for aggressive, treatment-resistant prolactinoma, as demonstrated by its efficacy in tumor shrinkage and normalization of hyperprolactinemia. In such complicated cases where treatment is limited, multidisciplinary collaboration is crucial for offering a holistic approach and achieving optimal outcomes for the patient.

## Data Availability

The datasets supporting the conclusions of this article are included within the article.

## Abbreviations

DA: Dopamine agonist
FSH: Follicular stimulating hormone
HPE: Histopathology examination
HPF: High-power field
MRI: Magnetic resonance imaging
PEG: Polyethylene glycol
RAPD: Relative afferent pupillary defect
TMZ: Temozolomide
TSH: Thyroid-stimulating hormone
TSS: Transsphenoidal surgery
VA: Visual acuity
